# Comparison of EV-free fraction, EVs, and total secretome of amniotic mesenchymal stromal cells for their immunomodulatory potential: a translational perspective

**DOI:** 10.3389/fimmu.2022.960909

**Published:** 2022-08-16

**Authors:** Andrea Papait, Enrico Ragni, Anna Cargnoni, Elsa Vertua, Pietro Romele, Alice Masserdotti, Carlotta Perucca Orfei, Patrizia Bonassi Signoroni, Marta Magatti, Antonietta R. Silini, Laura De Girolamo, Ornella Parolini

**Affiliations:** ^1^ Department of Life Science and Public Health, Università Cattolica del Sacro Cuore, Rome, Italy; ^2^ Fondazione Policlinico Universitario “Agostino Gemelli” Istituto di Ricovero e Cura a Carattere Scientifico, IRCCS, Rome, Italy; ^3^ Istituto di Ricovero e Cura a Carattere Scientifico, IRCCS Istituto Ortopedico Galeazzi, Laboratorio di Biotecnologie Applicate all’Ortopedia, Milan, Italy; ^4^ Centro di Ricerca E. Menni, Fondazione Poliambulanza Istituto Ospedaliero, Brescia, Italy

**Keywords:** amnion, secretome/conditioned medium, extracellular vesicles, mesenchymal stem/stromal cells, immune modulation

## Abstract

Amniotic mesenchymal stromal cells (hAMSCs) have unique immunomodulatory properties demonstrated *in vitro* and *in vivo* in various diseases in which the dysregulated immune system plays a major role. The immunomodulatory and pro-regenerative effects of MSCs, among which hAMSCs lie in the bioactive factors they secrete and in their paracrine activity, is well known. The mix of these factors (i.e., secretome) can be either freely secreted or conveyed by extracellular vesicles (EV), thus identifying two components in the cell secretome: EV-free and EV fractions. This study aimed to discern the relative impact of the individual components on the immunomodulatory action of the hAMSC secretome in order to obtain useful information for implementing future therapeutic approaches using immunomodulatory therapies based on the MSC secretome. To this aim, we isolated EVs from the hAMSC secretome (hAMSC-CM) by ultracentrifugation and validated the vesicular product according to the International Society for Extracellular Vesicles (ISEV) criteria. EVs were re-diluted in serum-free medium to maintain the EV concentration initially present in the original CM. We compared the effects of the EV-free and EV fractions with those exerted by hAMSC-CM *in toto* on the activation and differentiation of immune cell subpopulations belonging to both the innate and adaptive immune systems.

We observed that the EV-free fraction, similar to hAMSC-CM *in toto*, a) decreases the proliferation of activated peripheral blood mononuclear cells (PBMC), b) reduces the polarization of T cells toward inflammatory Th subsets, and induces the induction of regulatory T cells; c) affects monocyte polarization to antigen-presenting cells fostering the acquisition of anti-inflammatory macrophage (M2) markers; and d) reduces the activation of B lymphocytes and their maturation to plasma cells. We observed instead that all investigated EV fractions, when used in the original concentrations, failed to exert any immunomodulatory effect, even though we show that EVs are internalized by various immune cells within PBMC. These findings suggest that the active component able to induce immune regulation, tested at original concentrations, of the hAMSC secretome resides in factors not conveyed in EVs. However, EVs isolated from hAMSC could exert actions on other cell types, as reported by others.

## Introduction

Human amniotic mesenchymal stromal cells (hAMSCs) have attracted great attention thanks to their immunomodulatory properties (1, 2) that determine significant advantages for their application in the treatment of inflammatory or immune-mediated diseases (3). Indeed, hAMSCs exert strong antiproliferative and immunomodulatory actions on different immune cells. Several *in vitro* studies demonstrated that hAMSCs and their secretome inhibit the proliferation of T cells when activated through TCR stimulation. As a matter of fact, we previously reported that hAMSCs and their secretome prevent/reduce the development of cytotoxic T cells and the polarization toward inflammatory T helper subsets and enhance instead the polarization toward regulatory T cells (4, 5). Furthermore, they suppress the *in vitro* differentiation of monocytes toward inflammatory antigen-presenting cells, triggering instead the acquisition of phenotypical and functional features typical of M2 anti-inflammatory macrophages (5–8). We have also recently demonstrated that hAMSCs and their secretome are able to suppress the proliferation and differentiation of B lymphocytes to plasma cells (9).

The immunomodulatory properties of hAMSCs and their secretome have been shown to contribute to their ability to foster tissue regeneration and induce therapeutic effects in preclinical disease models. As a matter of fact, hAMSCs and their secretome have been successfully used to treat different inflammatory-related diseases including lung (10, 11) and liver fibrosis (12), wound healing (7, 13), collagen-induced arthritis (14), tendon-to-bone healing (15), bone defects (16), multiple sclerosis (14), inflammatory bowel disease (14), colitis (14, 17), sepsis (14), traumatic brain injury (18), and Huntington’s disease (19). All these studies strongly indicate that hAMSCs provide immunoregulatory actions through paracrine signaling triggered by factors present in their secretome, which include both freely secreted factors and factors conveyed by extracellular vesicles (EVs).

EVs are a heterogeneous family of bilayer membrane vesicles that, according to their size and biogenesis, are classified into exosomes (40–200 nm; generated within the endosomal system) and microvesicles (50–1,000 nm; produced by outward budding of the plasma membrane). EVs carry molecules such as proteins, lipids, and coding and non-coding RNAs, which can contribute to the function of the cells of origin from which they are released (20, 21). Some studies suggest that EVs released from MSCs may have a therapeutic action comparable to that of parental cells and their derived secretomes, thus representing an enormous therapeutic potential for applications in regenerative medicine due to their easier management/storage and higher safety (i.e., lack of tumorigenicity) compared to cell-based therapy (22). Our previous studies evidence that the hAMSC secretome (hereinafter defined as hAMSC-conditioned medium: hAMSC-CM) contains free, soluble molecules and EV-conveyed miRNAs with regulatory functions on different types of inflammatory cells (23). It is now crucial to define whether the EV fraction can exert biological activities comparable to those of the CM in toto. To this end, we compared the immunomodulatory potential of the different hAMSC secretome fractions. Indeed, no study has yet to specifically address the contribution that EVs and the freely secreted fraction have to the multifaceted immunomodulatory effects of hAMSC-CM. As a matter of fact, in this comparative study we aimed to discern the relative impact of the individual components of the hAMSC secretome, maintaining for each secretome fraction (EV-free and EV) the quantitative proportions in which they are represented in the secretome of origin.

Thus, herein for the first time we compared the immunomodulatory properties of the hAMSC secretome in toto with those of the different fractions (EV-free and EV fractions) to draw upon useful information for implementing future therapeutic approaches using immunomodulatory therapies (EV vs. secretome in toto) based on the MSC secretome.

## Material and methods

### Ethics statements

The collection of human peripheral blood mononuclear cells (PBMCs) for research purposes was approved by the Regional Departments of Transfusion Medicine (Rif. 523, July 7, 2016). PBMCs were obtained from healthy adult donors after informed consent and provided by the Center of Immune Transfusion of Spedali Civili of Brescia, Italy.

Human term placentae were collected from healthy women after vaginal delivery or caesarean section at term, after obtaining informed written consent, according to the guidelines set by the local ethical committee “Comitato Etico Provinciale di Brescia,” Italy (number NP 2243, 19 January 2016).

### Isolation of mesenchymal stromal cells from human amniotic membrane and CM preparation

Cells were isolated as previously described (24). The amniotic membrane was cut in fragments and digested at 37°C for 9 min with 2.5 U/ml dispase (VWR, Radnor, PA, USA). The digestion was then blocked by washing the amniotic fragments in RPMI complete medium composed of RPMI 1640 medium supplemented with 10% heat-inactivated fetal bovine serum (FBS), 1% penicillin/streptomycin (P/S), and 1% L-glutamine (all from Sigma-Aldrich, St. Louis, MO, USA). Enzymatic digestion continued with fragment incubation in the presence of 0.94 mg/ml collagenase and DNase I (both from Roche, Basel, Switzerland) for approximately 2.5–3 h at 37°C. The cell suspension obtained was centrifuged at low g, and the supernatant was filtered (100-μm cell strainer) (BD Falcon, Bedford, MA, USA), and the cells were collected by centrifugation. After isolation, hAMSCs (p0) were phenotypically characterized as previously reported (24). Cells with >80% expression of mesenchymal markers CD13 and CD90 and <10% expression of the hematopoietic marker CD45 and of the epithelial marker CD324 were used in this study.

Freshly isolated cells were expanded until passage 1 (p1) by plating at a density of 10^4^ cells/cm^2^ in Chang Medium C (Irvine Scientific, Santa Ana, CA, USA) supplemented with 2 mM L-glutamine at 37°C in the incubator at 5% CO2. To produce conditioned medium (CM), hAMSC p1 cells were seeded for 5 days in 24-well plates (Corning, NY, USA) at a density of 5 × 10^5^ cells/well in 0.5 ml of DMEM-F12 medium (Sigma-Aldrich) without serum, supplemented with 2 mM L-glutamine (Sigma-Aldrich) and 1% P/S as described (25).

At the end of the 5-day culture, CM was collected, centrifuged at 300×g, and filtered through a 0.2-μm sterile filter (Sartorius Stedim, Florence, Italy). An aliquot of 5 ml was kept frozen at − 80°C until use (CM in toto for comparison with other fractions), while the remaining CM volume (15–20 ml) was used for EV isolation. After EV isolation, the EV-free fraction was maintained for comparison studies. Six placentas were used to obtain six different hAMSC-CM preparations. The number of CMs used for each experiment is indicated in the figure legends.

### CM fractionation by ultracentrifugation

CM was centrifuged at 100,000 × g for 1, 3, 9, or 24 h at 4°C in a 70.1 Ti rotor (Beckman Coulter, Fullerton, CA, USA). To ensure optimal EV recovery, tubes were filled up to half nominal volume. After centrifugation, supernatants (EV-free fraction) were recovered and pellets (EV fraction) were suspended in a small volume of PBS or in a volume equivalent to the starting volume of CM used to isolate EVs (for example, EVs isolated from 12 ml of CM were resuspended in 12 ml of fresh medium without serum). Both EV-free and EV fractions were stored at 4°C and utilized within 24 h from isolation.

### EV detection by nanoparticle tracking analysis

EVs in the CM in toto, EV-free fractions, or EV fractions were visualized by the NanoSight NS300 system (NanoSight Ltd., Amesbury, UK). Dilutions with PBS were performed to ensure sample readings between 20 and 120 particles per frame. Pump flow was set to 30. Three to five recordings of 60 s were executed for each sample. Collected data were analyzed by the Nanoparticle Tracking Analysis (NTA) software, providing concentration measurements and high-resolution particle size distribution profiles.

### EV characterization by flow cytometry

For analysis of EV integrity, an identical number of EVs in both CM in toto and EV fraction samples calculated from NTA data were supplemented with carboxyfluorescein succinimidyl ester (CFSE; Sigma-Aldrich, St Louis, MO, USA) (10 μM final) and incubated in the dark at 37°C for 30 min. CFSE-positive events were detected and counted using the FITC channel up to a maximum value of 10,000 events per second with a 10-μl/min flow rate using a CytoFLEX flow cytometer (Beckman Coulter). Unstained CM in toto and EV fraction samples were used as negative controls to set CFSE positivity. Integrity was calculated as the ratio of FITC+ events between the EV fraction and CM in toto. Before analysis, flow cytometer performance in the nanometer range was set with reference Megamix-Plus SSC beads (Biocytex, Marseille, France) composed of FITC fluorescent spheres (160, 200, 240, and 500 nm).

For EV marker detection, CM in toto and EV fraction samples were stained with CFSE as previously described. Afterward, one-seventh of the labeled samples were stored at 4°C, whereas the rest was divided into six aliquots and each stained for 30 min at 4°C in the dark with 1 μl of the following APC-conjugated Ab: anti-CD9 (312107, BioLegend, San Diego, CA, USA), CD63 (353007, BioLegend), CD81 (349509, BioLegend), CD44 (338805, BioLegend), CD73 (344005, BioLegend), and CD90 (328113, BioLegend). Antibodies were used singularly. A maximum value of 10,000 events per second were acquired with a 10-μl/min flow rate using a CytoFLEX flow cytometer, and APC-positive events were detected after FITC gating as previously described. Unstained samples were used as negative controls to set APC positivity.

### EV characterization by transmission electron microscopy

EV fraction samples were suspended in PBS (100 μl per initial 5-ml volume). Five microliters was absorbed for 10 min at RT on formvar carbon-coated grids and drops blotted with filter paper. Two percent of uranyl acetate aqueous suspension was used to perform negative staining for 10 min, and excess was removed by filter paper. The grid was dried at RT. Samples were examined with a TALOS L120C transmission electron microscope (Thermo Fisher Scientific, Waltham, MA, USA) at 120 kV.

### Protein quantification in purified EVs

After centrifugation, samples from EV fractions were analyzed with a NanoDrop ND-1000 spectrophotometer (Thermo Fisher Scientific, Waltham, MA, USA) with the Protein A280 protocol. The concentration was related with NTA data to obtain the number of EVs/µg protein value.

### Analysis of T-cell proliferation

T-cell proliferation was induced by stimulating peripheral blood mononuclear cells (PBMCs) with an anti-CD3 monoclonal antibody. Human PBMCs were obtained from heparinized whole blood samples using density gradient centrifugation (Histopaque-1077, Sigma-Aldrich, St. Louis, MO, USA). PBMCs (1 × 10^5^/well in a 96-well-plate) were activated with 125 ng/ml (final concentration) anti-CD3 (Orthoclone OKT3, Janssen-Cilag, Cologno Monzese, Italy). Activated PBMCs (PBMC + anti-CD3) were cultured in the presence of different volumes of CM or of its fractions (EV-free or EVs) (10, 25, 50, or 100 μl/well of CM 5%, 12.5%, 25%, or 50%, respectively, of the final volume), for 3 days. The final volume of each well was 200 μl. In all experiments, activated PBMCs cultured alone were used as controls. All conditions were performed in triplicate in RPMI 1640 medium (Cambrex, Verviers, Belgium) supplemented with 10% heat-inactivated FBS, 2 mM L-glutamine, and P/S.

T-cell proliferation was assessed by 5-ethynyl-2′-deoxyuridine (EdU) incorporation as previously described (26). Briefly, 10 µM EdU (Life Technologies, Carlsbad, CA, USA) was added to PBMCs at day 3 post-stimulation. After 16–18 h, cells were harvested and EdU incorporation was evaluated by adding 2.5 μM 3-azido-7-hydroxycoumarin (Jena Biosciences, Jena, Germany) in buffer solution (100 mM Tris–HCl pH 8.0, 10 mM L-ascorbic acid, 2 mM CuSO_4_) at RT for 30 min. Cells were acquired using a FACSymphony A3 (BD Biosciences), and the percentage of proliferating EdU-positive cells was analyzed with FlowJo V10 (BD Biosciences). Cells were also stained with eFluor 780 (Thermo Fisher) for the exclusion of dead cells.

### Analysis of CD4^+^ T helper subsets and Treg subset polarization

Phenotypes were assessed by flow cytometry analysis of the expression of specific cell surface markers and transcription factors to identify T helper subsets (Th1, Th2, and Th17) and Treg. After 5 days of coculture in the presence of CM, PBMCs stimulated with anti-CD3 were collected and centrifuged at 300g for 5 min. Cells were stained with eFluor 780 for the exclusion of dead cells. The staining was performed using antibodies for CD3 (clone UCHT1), CD4 (clone VIT-4), CD45RA (clone HI100), CD196 (clone 11A9), CD183 (clone 1C6/CXCR3), CD25 (clone M-A25), and FoxP3 (clone 259D/C7) which all came from BD Biosciences and CD194 (clone REA279) from Miltenyi. Intracellular staining for FoxP3 was performed after fixation and permeabilization using BD Cytofix/Cytoperm (BD Biosciences), followed by staining with anti-FoxP3 antibody. Samples were acquired using a FACSymphony A3 and analyzed with FlowJo V10. T-cell subsets were identified by a sequential gating strategy. T effector cells were first identified by gating CD4-positive and CD45RA-negative cell populations (CD4^+^CD45RA^−^ cells), and different T helper (Th) subsets were identified as follows: Th1 as CD196^−^CD183^+^, Th17/Th1 as CD196+CD183+, and Th2 as CD196^−^CD183^−^CD194^+^ (27). Furthermore, to investigate the ability of hAMSC-CM and its fraction to induce Treg polarization, we performed a mixed lymphocyte reaction (MLR-T) by coculturing T lymphocytes (1 × 10^5^), previously isolated from PBMCs using the Pan T cell Isolation Kit (Miltenyi Biotech), with 1 × 10^5^ gamma-irradiated allogeneic PBMCs. Activated T lymphocytes (MLR-T) were cultured in the presence of different volumes of CM or of its fractions (EV-free or EVs) (10, 25, 50, or 100 μl/well of CM (5%, 12.5%, 25%, or 50%, respectively, of the final volume), for 6 days. Tregs were evaluated as previously reported in the aforementioned gating strategy, and the % of Treg cells was evaluated as CD25 High and FoxP3-positive cells.

### Analysis of monocyte differentiation toward antigen-presenting cells

Monocyte-derived M1 macrophages were differentiated starting from 5 × 10^5^ PBMCs cultured or not (control condition) in the presence of CM or its fractions, in 24-well plates (Corning) for 4 days in the presence of 5 ng/ml GM-CSF (Miltenyi Biotec) in 0.5 ml RPMI 1640 complete medium. Cells were activated by adding 20 ng/ml interferon gamma (IFN-γ) (Miltenyi Biotec) for 1 h after which 0.1 μg/ml LPS (Sigma-Aldrich) was added and left for 2 days. For dendritic cell (DC) differentiation, 2.5 × 10^5^ PBMCs were cultured in 48-well plates (Corning) for 4 days in the presence of 50 ng/ml recombinant human IL-4 (R&D Systems, Minneapolis, MN, USA) and 50 ng/ml GM-CSF in 0.5 ml RPMI 1640 complete medium. Complete maturation was reached by adding 0.1 μg/ml LPS for 2 days.

M1 macrophages, as well as DC, were collected after 6 days of differentiation in the absence or presence of 10, 25, 50, or 100 μl/well of CM or its fractions (EV-free or EVs) (2%, 5%, 10%, or 20%, respectively, of the final volume). Different CM products were added at day 0, concomitantly with the start of the differentiation protocol. Phenotype was investigated by flow cytometry. Prior to surface marker staining, cells were stained with eFluor 780 for the exclusion of dead cells. Cells stained with CD3 were excluded; CD11b (clone ICRF44)-positive cells were analyzed for the expression of CD163 (clone GHI/61), CD197 (clone 3D12), CD86 (clone 2331 (FUN-1)), and CD14 (clone MΦP9) to distinguish M1/M2 macrophage polarization. For mDC differentiation, the same protocol aforementioned for the macrophages was used and staining was performed for CD197 (clone 3D12), CD209 (clone DCN46), CD14 (clone MΦP9), CD83 (clone HB15e), and CD1a (clone HI149). All antibodies were purchased from BD Biosciences.

### Analysis of B-cell proliferation and differentiation

To evaluate B lymphocyte proliferation, PBMCs were labeled with CFSE to monitor cell division (9). Stained PBMCs (1 × 10^5^ cells) were seeded in 96-well tissue culture plates in 50% RPMI complete medium and 50% UltraCULTURE™ serum-free culture medium (Lonza, Basel, Switzerland). B-cell proliferation was induced by stimulating cells for 6 days with 2.5 μg/ml CpG-ODN 2006 (Aurogene s.r.l., Rome, Italy). Activated PBMCs were cultured in the presence of CM-hAMSC or its fractions, EV-free, or EVs (10, 25, 50, or 100 μl/well of CM 5%, 12.5%, 25%, or 50%, respectively, of the final volume).

Cells were stained with eFluor 780 for the exclusion of dead cells. The staining was performed using antibodies for CD3 (clone UCHT1), CD19 (clone SJ25C1), CD14 (clone MΦP9), CD27 (clone M-T271), CD24 (clone ML5), CD38 (clone HB7), and IgM (clone G20-127) which all came from BD Biosciences and CD138 (clone 44F9) from Miltenyi. Samples were acquired using a FACSymphony A3 and analyzed with FlowJo V10. B-cell subsets were identified by a sequential gating strategy. Plasmablasts were identified as CD19^+^CD27^hi^CD38^hi^CD138^-^ cells; plasma cells (PC) were identified by the co-expression of the CD138 marker (CD19^+^CD27^hi^CD38^hi^CD138^+^).

### Quantification of hAMSC-EV uptake by PBMC

The spontaneous uptake of EVs by PBMC was analyzed by culturing unstimulated PBMC for 18 h with 5 × 10^9^ vesicles stained with CFSE, as previously reported in the section entitled “EV characterization by flow cytometry”. After incubation, the PBMCs were collected, extensively washed, and analyzed by flow cytometry for uptake. To evaluate the uptake ability of the different immune cell subsets, we stained the PBMCs with the following antibodies: CD3 (clone UCHT1), CD19 (clone SJ25C1), and CD14 (clone MΦP9). The amount of immune cells positive for the expression of CFSE and indicative of the EV uptake was assessed with FACSymphony A3 and analyzed with FlowJo V10. The comparison was performed using untreated PBMC as baseline for basal autofluorescence.

### Statistical analysis

The data are shown as violin-truncated plots with Tukey variations. The parameters were compared using one-way and two-way analyses of variance (ANOVA). Data are representative of at least three independent experiments. Statistical analysis was performed using Prism 8 (GraphPad Software, La Jolla, CA, USA). A *p* value lower than 0.05 was considered statistically significant.

## Results

### hAMSC-CM fractionation

**Figure 1:**
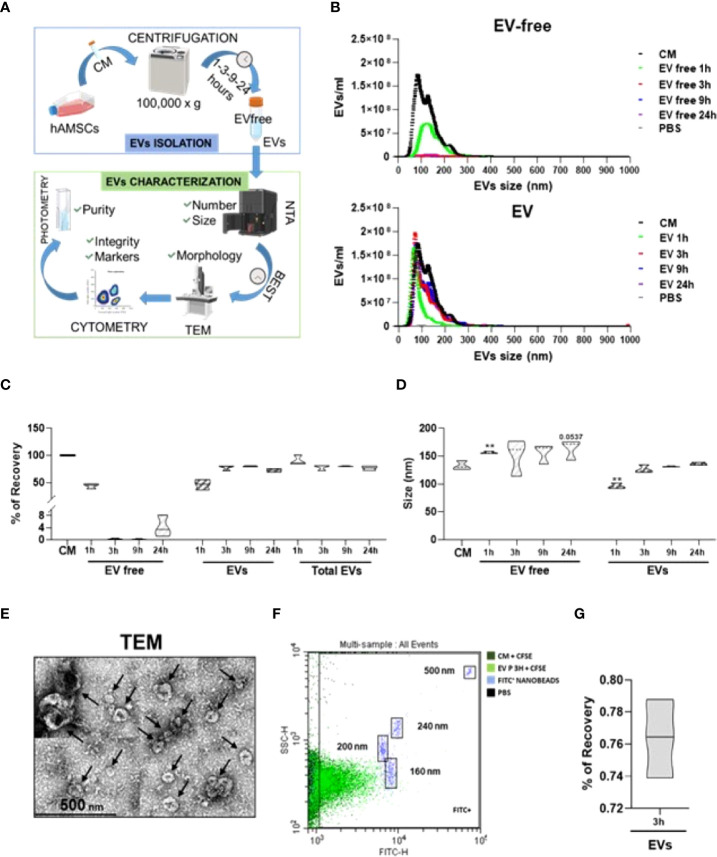
Definition of hAMSC-CM fractionation protocol and EV characterization. **(A)** Workflow of the protocol used to identify and validate the most efficient centrifugation procedure to obtain EV-depleted and EV-enriched fractions from hAMSC-CM. **(B)** Particle size and concentration profiles from NTA data for both fractions (EV free and EVs) after centrifugation for the indicated times (1, 3, 9, and 24 h). CM and PBS were used as starting condition and background, respectively (N = 3, values indicated as mean ± SD). **(C)** EV recovery or contamination in the EV-free fraction for the tested centrifugation times (1, 3, 9, and 24 h) calculated from the NTA data with respect to EVs in the CM set as 100% (N = 3, mean ± SD). **(D)** Size analysis from NTA data for the EVs identified in the CM, EVs, or EV-free fractions obtained after centrifugation for the indicated times (1, 3, 9, and 24 h) (N = 3, mean ± SD). **(E)** Transmission electron micrographs of EVs in the 3-h centrifugation pellets showing characteristic cup-shaped morphology and size compatible with NTA data. Black arrows indicate the EVs. **(F)** Visualization of EVs (in the unprocessed CM or in EV-fraction) after a 3-h centrifugation), after CFSE staining and comparison with nanometric FITC-fluorescent beads of indicated sizes. A representative cytogram is presented. **(G)** % of EVs recovery after 3 h of centrifugation.

In order to efficiently isolate EVs from the hAMSC-CM and obtain the EV-depleted counterpart, a tailored protocol was fine-tuned relying on a centrifugal force of 100,000 × g, as widely reported in the ISEV guidelines as an efficient method for EV isolation from CM (28). This was followed by several quality controls (**Figure 1A**). On a pilot hAMSC-CM lot (15.3 × 10^9^ ± 0.4 EVs/ml), different centrifugation times were scored to identify via NTA technology the experimental condition coupling the highest EV removal and recovery with the most reduced operative time to avoid EV damage by long-lasting g-force (**Figure 1B**). The combination of 100,000 × g and 3-h centrifugation resulted in the best protocol with EV recovery up to 77.9% ± 4.2 (**Figure 1C**). Notably, increasing the centrifugation times did not allow for higher recovery (80.4% ± 0.8 and 72.6% ± 2.8, 9 and 24 h, respectively), whereas a shorter time (1 h) did not lead to efficient separation of particles from the supernatant that resulted to be contaminated (44.0% ± 4.0 of the initial EVs input with 46.6% ± 7.9 recovery). Moreover, the selected 3-h protocol led to the isolation of particles of comparable size with respect to EVs in the CM (133 nm ± 8 vs. 127 nm ± 7, CM vs. EVs, no significant (p < 0.05) difference) (**Figure 1D**), as scored by NTA data, while 1-h centrifugation resulted in particles of smaller size (96 nm ± 5), possibly due to the selection of a subpopulation as clearly evidenced in NTA plots where larger particles are mainly detected in the supernatant. NTA graphs also showed the absence of larger (>300 nm) events both before and after EV isolation, emphasizing that the procedure does not lead to particle aggregates or clumps, even after 24 h of centrifugation. Size was substantiated by transmission electron microscopy (TEM) analysis of isolated EVs, showing their typical cup-shaped morphology, with the majority of particles below 200 nm and few large ones (>250 nm) as reported by NTA (**Figure 1E**). The isolation of particles with dimensional parameters similar to those in the CM suggests the conservation of EV integrity after processing, a crucial step in order to subsequently attribute a biological effect. By flow cytometry analysis of CFSE-stained particles, compared to those in the unpurified CM, and by direct comparison with fluorescent nanobeads of predetermined size (160, 200, 240, and 500 nm), the dimensional range previously obtained with NTA technology was further confirmed to be around 150 nm (**Figure 1F**) both before and after purification of another parameter that confirmed the absence of major particle disruption or aggregation. Finally, we reported that EV integrity after centrifugation showed 76.4% ± 2.0 (**Figure 1G**). Flow cytometry also showed the low and high presence of CD9 and CD63/CD81 EV markers, respectively, as well as both the abundance of CD73/90 and the whole population shift of CD44 hAMSC markers (**Supplementary Figure 1**), as previously published by our group (23). Comparable data before and after centrifugation were a further support of absence of detrimental effects or isolation of subpopulation given by the isolation procedure. As a final quality control, the purity of isolated EVs was calculated and resulted to be 0.468 × 10^9^ ± 0.095 EVs/μg protein, a value falling within the range (10^8^ to 10^10^) reported for EV preparations from cell culture supernatants (29).

### Characterization of EVs from hAMSC-CM

To try to discriminate the impact of the different components of the secretome, we compared the results obtained from the secretome in toto (hAMSC-CM) with those of the same secretome depleted from EVs (EV-free) and with the EV fraction resuspended in an equivalent volume of fresh medium. Specifically, in order to evaluate the effect due to EVs alone and discern it from that of soluble bioactive molecules contained in the EV-depleted secretome, we suspended EVs in the equivalent volume of fresh medium (DMEM-F12) (for example, EVs isolated from 12 ml of CM were resuspended in 12 ml of fresh medium). The products obtained were subsequently characterized as previously described (**Table 1**). The average EV concentration per ml resulted to be 12.0 × 10^9^ ± 7.6 in CM, 2.0 × 10^9^ ± 2.9 in EV-free, and 9.9 × 10^9^ ± 5.5 in EV fractions.

**Table 1:** Summary table for concentration, recovery, size, and marker expression of EVs isolated from hAMSC-CM.

EV/ml (×10^8^) (N = 6)		
	MEAN	SD
CM-hAMSC	12.0	7.6
EV-free	2.0	2.9
Evs	9.9	5.5
**Recovery (%) (N = 6)**		
	**MEAN**	**SD**
EV-free	12.9	10.9
Evs	85.8	12.8
**Size (nm) (N = 6)**		
	**MEAN**	**SD**
CM-hAMSC	149	21
EV-free	153	17
Evs	147	17
**Markers (%) (N = 3)**		
EV-CD9	5	1
EV-CD83	88	1
EV-CD81	87	2
EV-CD44	40	9
EV-CD73	90	2
EV-CD90	81	1
**Purity (EV × 10^8/^µg) (N = 4)**		
	**MEAN**	**SD**
EVs	0.178	0.06

Overall, the recovery rate was similar to the values previously observed during protocol optimization resulting to be 85.8% ± 12.8. Mean EV size was calculated in 149 nm ± 21 and 147 nm ± 17 for CM and EV samples, respectively, with no significant (p < 0.05) difference between conditions. On three random EV samples, we monitored the presence of EV and hAMSC markers obtaining results almost identical to those previously observed with a very weak presence of CD9 (5% ± 1), abundant signal for CD63 (88% ± 1), CD81 (87% ± 2), CD73 (90% ± 2), and CD90 (81% ± 1), and weaker staining albeit with a whole population shift for CD44 (40% ± 9). Also, purity on four samples resulted to be 0.178 × 109 ± 0.060 EVs/μg protein. Analysis on a single sample showed 78% EV integrity. Given the reliability of the procedure, in all experimental conditions discussed in the following paragraphs, with exception when indicated, we compared the effect of a range of comparable doses of hAMSC-CM and EV-free and EV fractions (100, 50, 25, and 10 µl).

### Ability of hAMSC-CM and EV-free and EV fractions to modulate T-cell proliferation

**Figure 2:**
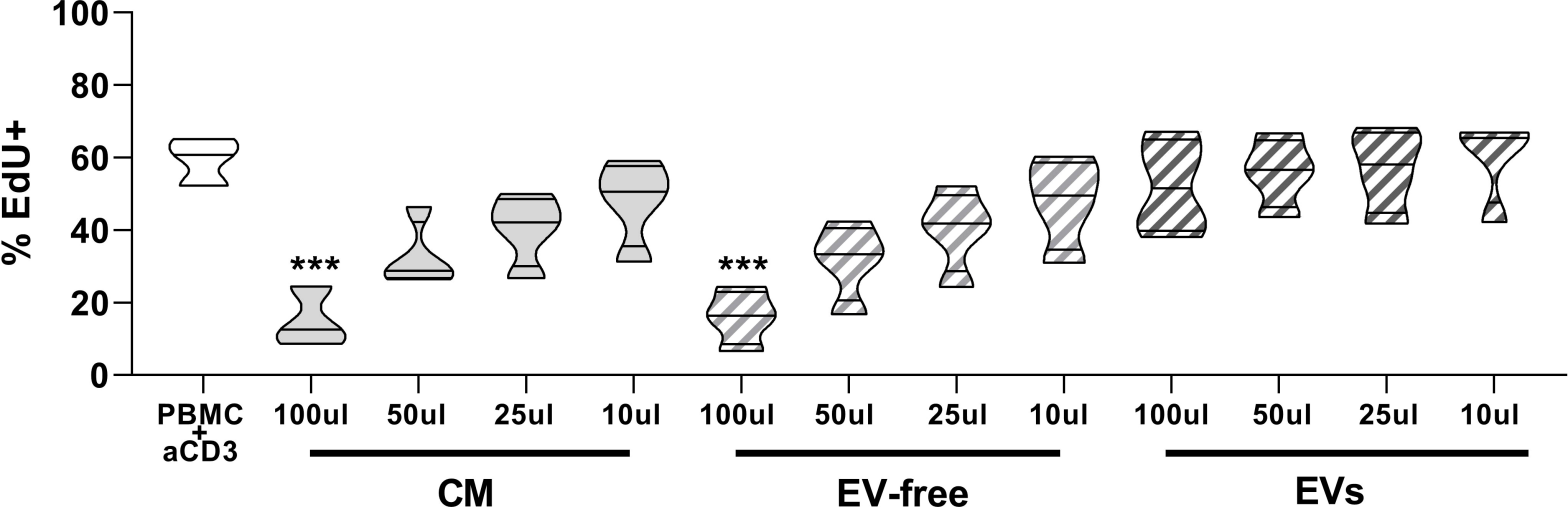
Effects of hAMSC-CM and its fractions (EV-free and EVs) on PBMC proliferation. Allogeneic PBMC (1 × 10^5^) were stimulated with anti-CD3 antibody in the presence of 100, 50, 25, or 10 µl of hAMSC-CM *in toto* or with the same volume of the EV-free or EV fractions. Results are expressed as percentage of EdU+ cells representative of the proliferating cells. PBMCs stimulated with anti-CD3 mAb constitute the positive control. Results are displayed as violin plots showing median (thick line) and 25th and 75th quartiles (***p < 0.001 versus control (PBMC + anti-CD3), N ≥ 3.

First, we evaluated and compared the effects of different doses of hAMSC-CM in toto and fractions obtained thereof (EV-free and EV fractions) on the proliferation of T lymphocytes stimulated with anti-CD3. The results obtained confirmed that CM in toto was able to inhibit the proliferation of T lymphocytes in a dose-dependent manner (from 59.4 ± 6.6% to 15.2 ± 8.3% of proliferating T cells in control and in activated PBMCs treated with 100 µl of CM, respectively; p < 0.001) and that only at the lowest dose (10 µl) was this effect partially lost (**Figure 2**). A similar trend was appreciable for the EV-free fraction (15.9 ± 7.5% proliferating cells in activated PBMCs treated with 100 µl of EV-free fraction, p < 0.001) (**Figure 1**), whereas no appreciable effect was observed for EVs at any dose used (52.1 ± 13.1% proliferating cells in activated PBMCs treated with 100 µl of EVs, *p*=ns).

### Ability of hAMSC-CM and EV-free and EV fractions to modulate Th subsets and Treg polarization

**Figure 3:**
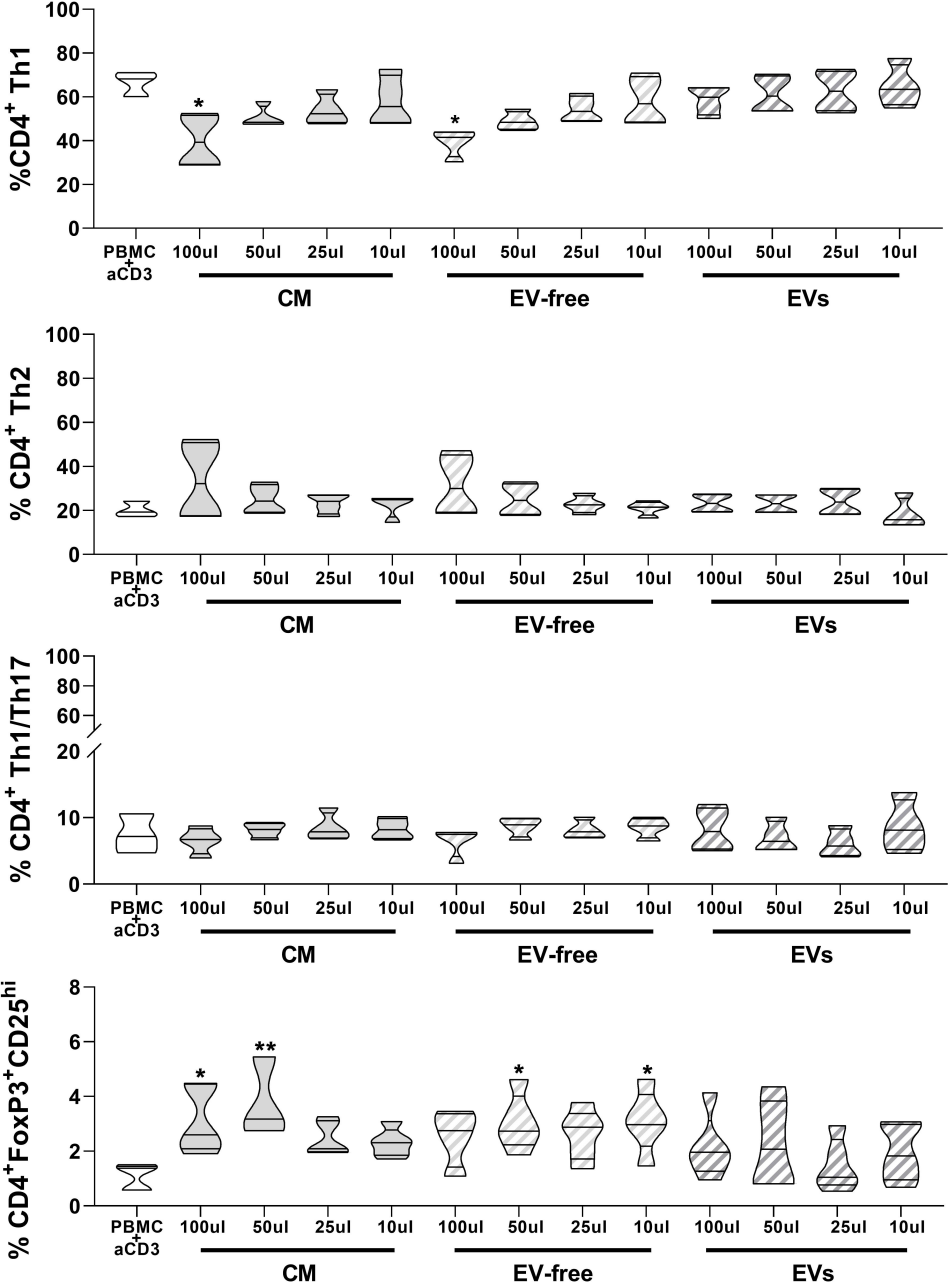
Effects of hAMSC-CM and its fractions (EV-free and EVs) on the differentiation of T lymphocytes toward Th1, Th2, Th1/Th17, and Treg subtypes. Allogeneic PBMCs were incubated with anti-CD3 mAb in the presence of 100, 50, 25, or 10 µl of hAMSC-CM in toto or with the same volumes of the EV-free or EV fractions. Samples were collected after 5 days of culture. Results are expressed as percentage of the different Th subsets investigated: Th1 (CD183^+^CD196^−^), Th2 (CD183^-^CD196^-^CD194^+^), Th1/Th17 (CD183^+^CD196^+^), and Treg (CD4^+^CD25^hi^FoxP3^+^). Results are displayed as violin plots showing median (thick line) and 25th and 75th quartiles (*p < 0.05, **p < 0.01, N ≥ 3 individual experiments).

Given that EVs are carriers of miRNAs and given the importance that miRNAs have on modulating the cell differentiation process (30), we investigated the impact of hAMSC-CM and its fractions on the differentiation of effector T cells toward various Th subsets. This study was performed, as done for T-cell proliferation analysis, by stimulating PBMCs with anti-CD3 monoclonal antibody in the presence of hAMSC-CM in toto or of its two fractions (EV-free or EVs). The commitment toward the different Th subsets was evaluated by flow cytometry, and the results were expressed as % of CD4-positive cells expressing specific markers identifying Th1, Th2, or Th1/Th17 subsets. As shown in **Figure 3**, hAMSC-CM decreased the median percentage of Th1 in PBMC stimulated with anti-CD3 with respect to control without CM treatment (40.0 ± 12.3% vs. 66.5 ± 5.7% in activated PBMC treated with 100 µl CM and in control activated PBMCs, respectively; *p* < 0.05). A similar trend was observed for the EV-free fraction (39.3 ± 6.3% in activated PBMCs treated with 100 µl of EV-free fraction, *p* < 0.05). When anti-CD3 stimulated PBMCs were cocultured in the presence of the EV fraction, we observed a slight reduction in Th1 median percentage (58.6 ± 6.6% in activated PBMCs treated with 100 µl EV fraction, *p*=ns), but this reduction was not statistically significant.

No significant differences were observed for Th2 polarization or the generation of Th1/Th17 subsets, for either hAMSC-CM in toto or the different fractions tested.

In addition, we investigated the effects of the hAMSC-CM in toto or its fractions (EV-free or EVs) on Treg subset polarization (**Figure 3** lower panel). As previously reported, we confirmed that CM-hAMSC triggers the induction of Treg cells at the highest concentration (1.08 ± 0.46% vs. 3.34 ± 1.04%, respectively). Similar results were also obtained by the EV-free fraction at the intermediate and lowest concentrations (50 and 10 µl: 3.046 ± 1.03% and 3.094 ± 1.14%, respectively). Also in this case, the EV fraction seems to be able to induce only a slight increase in the Treg subset polarization at the highest concentration used (2.14 ± 1.20%).

### Ability of hAMSC-CM and EV-free and EV fractions to modulate monocyte differentiation to antigen-presenting cells

Our group previously demonstrated that hAMSC-CM hampers monocyte (Mo) differentiation toward M1-type macrophages and instead triggers the acquisition of anti-inflammatory M2 macrophage features (6). Here we wanted to clarify the impact that the EV-free fraction and EVs have on Mo-M1 differentiation comparing the results with those obtained with hAMSC-CM in toto.

**Figure 4:**
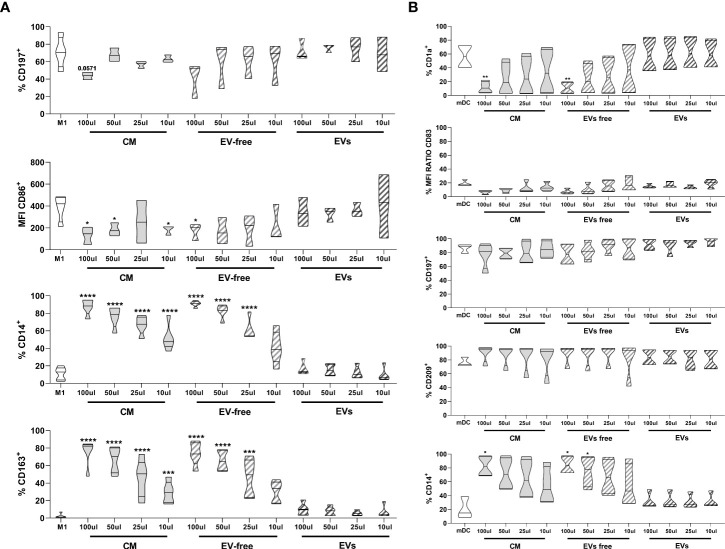
Effects of hAMSC-CM and its fractions (EV-free and EVs) on monocyte differentiation toward antigen-presenting cells **(A)** M1 macrophages were obtained by incubating PBMCs with GM-CSF for 4 days, followed by IFN-γ + LPS for other 2 days. At the end of the culture period, the expression of CD197 and of the co-stimulatory molecule CD86, together with the pro-monocytic marker CD14, was evaluated by flow cytometry for M1 macrophages. The M2 polarization was assessed by analyzing the expression of the CD163 marker. **(B)** mDC differentiation was carried out by incubating PBMCs with GM-CSF + IL-4 for 4 days followed by 2 days of LPS treatment. At the end of the culture period, expressions of CD1a, CD197, CD209, CD14, and the co-stimulatory molecule CD83 were evaluated by flow cytometry for mDC differentiation. Results are presented as a percentage of expression or mean fluorescence intensity (MFI) ratio (between MFI control and MFI treated samples) and are shown as violin plots with median (thick line) and 25th and 75th quartiles (*P < 0.05, **P < 0.01, ***P < 0.001, ****P < 0.0001 versus control M1 **(A)** or mDC **(B)**, N ≥ 3 individual experiments.

Both hAMSC-CM as well as the EV-free fraction reduced monocyte differentiation toward M1 macrophages, both by maintaining the expression of the undifferentiated monocytic marker CD14 (11.2 ± 7.2% control Mo-M1 macrophages vs. 87.8 ± 8.85% hAMSC-CM 100 µl and 90.9 ± 3.34% EV-free fraction 100 µl) (**Figure 4A**) and by reducing the expression of markers associated with M1 differentiation, such as CD197 (chemokine receptor CCR7) (70.8 ± 18.2% control Mo-M1 macrophages vs. 43.7 ± 4.30% hAMSC-CM 100 µl and 41.6 ± 20.5% EV-free fraction 100 µl) and the co-stimulatory molecule CD86 (MFI 384.8 ± 122.8 control Mo-M1 macrophages vs. 133.7 ± 80.2 hAMSC-CM 100 µl and 171.4 ± 78.5% EV-free fraction 100 µl) (**Figure 4A**). In parallel, they promoted monocyte skewing toward alternatively activated (M2) macrophages, as indicated by the significant increase in the percentage of macrophages expressing the M2 marker CD163 (all the results refer to the 100-µl concentration: 2.29 ± 2.64% control Mo-M1 macrophages vs. 75.3 ± 15.4% hAMSC-CM and 73.8 ± 13.4% EV-free fraction). On the other hand, the EV fraction did not affect monocyte differentiation toward M1 macrophages. Indeed, no differences vs. control Mo-M1 macrophages were observed with the administration of EVs regardless of the concentration used (all the results refer to the 100-µl concentration: CD14 (16.76 ± 7.21%), CD197 (72.2 ± 12.65%), CD86 (MFI 341.97 ± 131.4), and CD163 (9.6 ± 7.14%) (**Figure 4A**).

In parallel, we investigated the ability of CM-hAMSC in toto and the two fractions to impact monocyte differentiation toward myeloid dendritic cells (mDC) (**Figure 4B**), while both the CM in toto and the EV-free fraction were able to impact monocyte-derived mDC (Mo-mDC) differentiation by lowering the expression of the maturation marker CD1a (56.5 ± 18.01% control Mo-mDC vs. 11.8 ± 8.45% hAMSC-CM 100 µl and 10.9 ± 8.3% EV-free fraction 100 µl) and of the costimulatory molecule CD83 (MFI 18.8 ± 4.14 control Mo-mDC vs. 6.5 ± 2.67 hAMSC-CM 100 µl and 7.2 ± 3.77 EV-free fraction 100 µl), with a slight effect on CD197 expression (86.4 ± 6.6% control Mo-mDC vs. 76.5 ± 18.3% hAMSC-CM 100 µl and 84.87 ± 12.14% EV-free fraction 100 µl). No differences were observed in the expression of the dendritic marker CD209 (76.6 ± 6.87% control Mo-mDC vs. 90.12 ± 12.5% hAMSC-CM 100 µl and 88.75 ± 14.25% EV-free fraction 100 µl). Furthermore, we confirmed the ability of CM-hAMSC to impair the monocyte differentiation fostering instead the maintenance of the promonocytic marker CD14 (20.5 ± 15.9% control Mo-mDC vs. 82.8 ± 14.76% hAMSC-CM 100 µl and 84.3 ± 13.02% EV-free fraction 100 µl). On the other hand, also for this subset no differences were attributable to the exogenous administration of the EVs regardless of the concentration used (all the results refer to the 100-µl concentration: CD1a (58.5 ± 25.7%), CD83 (MFI 15.03 ± 3.07), CD197 (91.6 ± 7.82%), CD209 (9.6 ± 7.14%), and CD14 (31.17 ± 11.76%) **Figure 4B**.

### Ability of hAMSC-CM and EV-free and EV fractions to modulate B lymphocyte proliferation and differentiation toward antibody-secreting cells

In order to provide a comprehensive analysis of the immunomodulatory properties of hAMSC-CM and its fractions, we also evaluated their effects on B lymphocyte proliferation induced by PBMC stimulation with CpG.

B-cell proliferation and differentiation were evaluated by flow cytometry, and specifically by the percentage of CD19+-proliferating cells, and the expression of markers specific for B-cell differentiation toward antibody-secreting cells and specifically plasmablasts (CD19^+^CD27^High^CD38^High^CD138^−^ cells) and plasmacells (CD19^+^CD27^High^CD38^High^ CD138^+^ cells).

**Figure 5:**
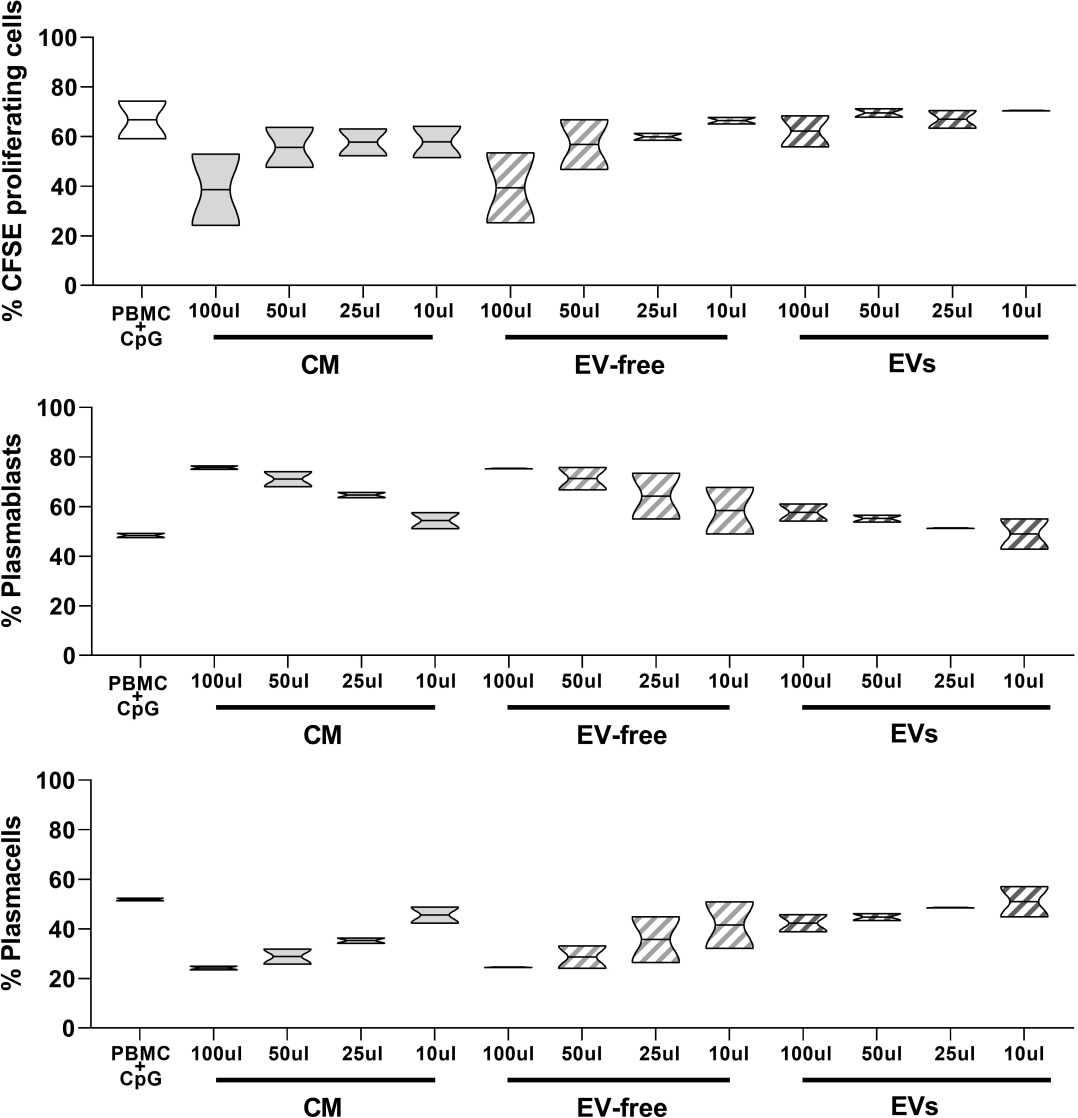
Effects of hAMSC-CM and its fractions (EV-free and EVs) on B lymphocyte proliferation and maturation. Allogeneic PBMCs (1 × 105) were stimulated with CpG in the presence of 100, 50, 25, or 10 µl of hAMSC-CM or with the same volume of the EV-free or EV fractions. B-cell proliferation was measured by the analysis of CFSE dilution and calculated as percentage of CD19+-proliferating cells. The impact that hAMSC-CM and its fractions have on B lymphocyte differentiation was evaluated analyzing the total amount of plasmablasts (CD19^+^CD27^hi^CD38^hi^CD138^-^) and plasma cells (CD19^+^CD27^hi^CD38^hi^CD138^+^) obtained from the different culture conditions and compared with the control condition. Results are presented as a percentage of expression and are shown as violin plots with median (thick line) and 25th and 75th quartiles versus control PBMC+CpG, N ≥ 2 individual experiments.

As shown in **Figure 5**, upper panel, the percentage of proliferating CD19^+^ cells in the control condition represented by untreated PBMC activated with CpG (PBMC+CpG) was strongly reduced in the presence of hAMSC-CM (100 µl) (66.8 ± 10.74% to 38.6 ± 20.36%, respectively). When we analyzed the effect of the EV-free fraction, we observed results similar to those obtained with hAMSC-CM. The EV-free fraction (100 µl) indeed decreased the percentage of CD19^+^-proliferating cells to 39.4 ± 19.9%. Finally, EVs induced a slight reduction of CD19+-proliferating cells in comparison to hAMSC-CM and the EV-free fraction (62.2 ± 8.9%).

Moreover, we observed a higher percentage of plasmablasts (**Figure 5**, middle panel) in the presence of hAMSC-CM (and consequently a lower percentage of plasma cells), with respect to that observed in the control PBMCs stimulated in the absence of hAMSC-CM (48.4 ± 1.27% vs. 75.75 ± 1.06%, respectively). We obtained comparable results when stimulated PBMCs were exposed to the EV-free fraction (75.45 ± 0.2%), while no differences were observed when we performed the test in the presence of EVs.

Conversely, we observed that the percentage of plasma cells was strongly increased in the control condition with respect to the PBMCs activated in the presence of hAMSC-CM and the EV-free fraction (51.9 ± 0.8% to 24.2 ± 1.06% and 24.5 ± 0.2%, respectively), while only small differences were appreciable in the presence of EVs (42.3 ± 4.94%) (**Figure 5** lower panel).

### Uptake of hAMSC-EVs by immune cells

**Figure 6:**
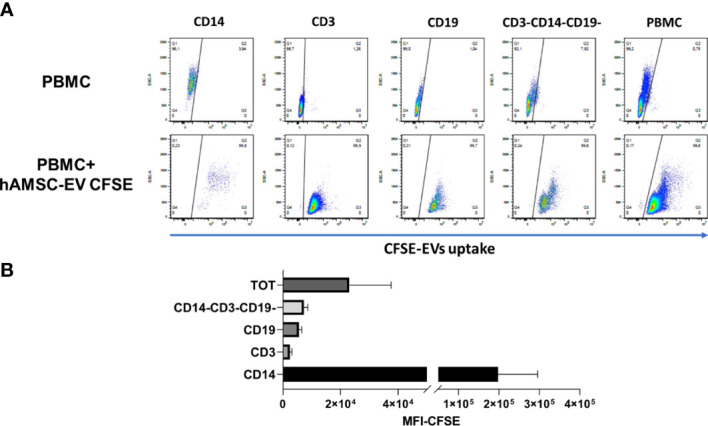
Flow cytometry analysis of hAMSC EV uptake by PBMC **(A)** PBMC were cultured without stimulation in presence or absence of 5 × 10^9^ EVs loaded with CFSE to evaluate uptake. A staining for the different immune subsets was performed to evaluate differences in the uptake capability. **(B)** Mean fluorescence analysis of the different immune subpopulations normalized on the negative fraction.

In an effort to exclude the lack of uptake by PBMCs as a possible mechanism responsible for the negligible immunomodulatory effect of EVs, we performed an uptake experiment by labeling EVs with CFSE. The CFSE-labeled vesicles were added to the unstimulated PBMC. We chose to evaluate fluorescence-labeled exosome uptake under a condition that could be considered similar for all immune PBMC cell types, and thus we considered the quiescent condition since activation conditions can vary between different types of immune cells. After 18 h, whole PBMC and major immune populations were analyzed by flow-cytometry to evaluate CFSE-positive cells. We observed that PBMCs were 99.8% positive for CFSE staining (**Figure 6**), indicating that EVs were taken up by immune cells. Furthermore, we observed that among the different immune populations, CD14^+^ cells, representative of the monocyte compartment, were highly positive for CFSE expression in comparison to the lymphocyte compartment, considering both T and the B lymphocytes ((CD3^-^CD19^+^ cells), respectively).

## Discussion

Our previous studies evidenced the strong immunomodulatory activities of mesenchymal stromal cells derived from amniotic membrane (hAMSC) and the essential role of the secreted bioactive factors in mediating these activities.

Given that these factors can be directly secreted as free molecules or conveyed in EVs, in this study we explored the contribution of all these components to the immunomodulatory activity of the hAMSC secretome. To this end, we compared the ability of the secretome in toto versus EV-free and EV fractions to affect immune cell subsets *in vitro*.

Our study deeply investigated the effects of the hAMSC secretome and its fractions on immune cells and was not limited to selected immune populations (31, 32) but included immune cells belonging to both innate and adaptive immune systems.

For the first time, EV and EV-free fractions were compared with the secretome in toto by maintaining the quantitative proportions as the secretome of origin. These study conditions allowed us to depict a reliable representation of the relative immunomodulatory potency of each secretome component.

Our data evidenced that the ability of the hAMSC secretome to modulate the immune cell response is mainly mediated by factors non-conveyed by EVs. In fact, the EV-free fraction, comparable to the secretome in toto, inhibited T-cell proliferation, reduced T-cell polarization toward the Th1 subset, and promoted the induction of Treg. In addition, the EV-free fraction, similar to the secretome in toto, maintained the ability to shift monocyte differentiation toward M2 instead of M1 macrophages, to reduce the maturation of dendritic cells and to reduce the CpG-induced B-cell proliferation and differentiation toward plasmablasts and plasmacells. The EV fraction instead did not affect any of these immune parameters despite that hAMSC EVs are taken up by all types of immune cells investigated.

Our results are in line with those published by Lange-Consiglio and colleagues (33) that showed that EVs derived from equine amniotic MSCs were not able to affect PBMC proliferation. However, no other study except ours has investigated the effects of EVs from hAMSC on a wide spectrum of immune cells.

EVs from other types of MSCs, including MSCs from the bone marrow (BM), umbilical cord (UC), and adipose tissue (AT), were investigated for their ability to control the proliferation of different immune cells among which are T cells, B cells, and NK cells, but with controversial results (31, 32, 34–37). For example, some studies have shown an immunosuppressive action of EVs on T cells (34, 38); other studies instead found lower effects of EVs in relation to parental MSC (31, 32, 36), while some demonstrated no effect at all of EVs (35, 39). Controversial results were also obtained by studies focusing on the effect of MSC-derived EVs on B cells (31, 37, 40).

These different findings can potentially derive from some methodological limitations that make the various studies incomparable. For example, different methods of EV quantification were applied, such as protein concentration or EV absolute counts by NanoSight, making it difficult to identify the possible effective dose. To this regard, we explored the effects of three increasing doses of EVs (10, 50, and 100 µl) corresponding to a wide range of EV absolute number (from 1.8 × 10^8^ to 2.0 × 10^9^ vesicles). This range covers and even exceeds the EV doses used in most of the reported studies: 5–100 × 10^6^ EVs (33), 3 × 10^6^ EVs/10^4^ immune cells (31), and 0.75 and 3 × 10^9^ particles/ml (41) and 10^9^ particles (34). Furthermore, to exclude the possibility that the lack of effect observed for hAMSC-EVs was due to a low concentration of EVs, we repeated the immunomodulatory tests using twice the concentration of EVs used in the tests (i.e., 200 µl instead of 100 µl); however, we still did not observe any effect on the different immune populations tested (data not shown). Although the EV-free fraction contained a residual amount of EVs, which represents an unavoidable compromise to obtain the highest EV removal avoiding EV damage, it is very unlikely that the residual EV amount can be responsible for the immunomodulatory activities observed for the EV-free fraction; otherwise, we would have observed some of these activities also for the EV fraction.

Another important factor that could heavily impact functions of MSC-derived EVs is the MSC culture conditions used to produce CM from which EVs are isolated. The extracellular microenvironment indeed affects the composition of EVs and the consequent biological activities (42). Likewise, different methodologies have been used to isolate EVs with different impact on EV biological functions (43, 44). For example, it has been recently reported (45) that contaminating soluble factors can increase the apparent bioactivity of EV even if the specific role on immunomodulatory properties has not been elucidated.

Moreover, it is to be considered that a given type of cell (i.e., MSC) may secrete heterogenous types of EVs characterized by differential exosome content and different subsets of exosomes with distinct proteomic profiles suggesting distinct biological functions (46–48). Thus, a specific function cannot be generalized to all MSCs and to all EVs/exosomes secreted from these cells.

Although we found that hAMSC EVs play a negligible role in determining the immunomodulatory properties of the hAMSC secretome, others have demonstrated that they exert *in vivo* beneficial effects on animal models of inflammation-driven diseases such as chronic endometritis (49), liver fibrosis, non-alcoholic steatohepatitis (50), and osteoarthritis (51). Furthermore, *in vitro* studies demonstrated that hAMSC EVs are able to reduce injury/activation in endometrial cells (52), in hepatic stellate cells (50), and in tenocytes (33). These findings, together with our recent unpublished results, that demonstrate the ability of hAMSC EVs to promote the expansion and the differentiation of dystrophic progenitor muscle cells suggest that hAMSC EVs might preferentially target injured organ/tissue specific cells by directly promoting their integrity and survival and differentiation.

## Conclusion

**Figure 7:**
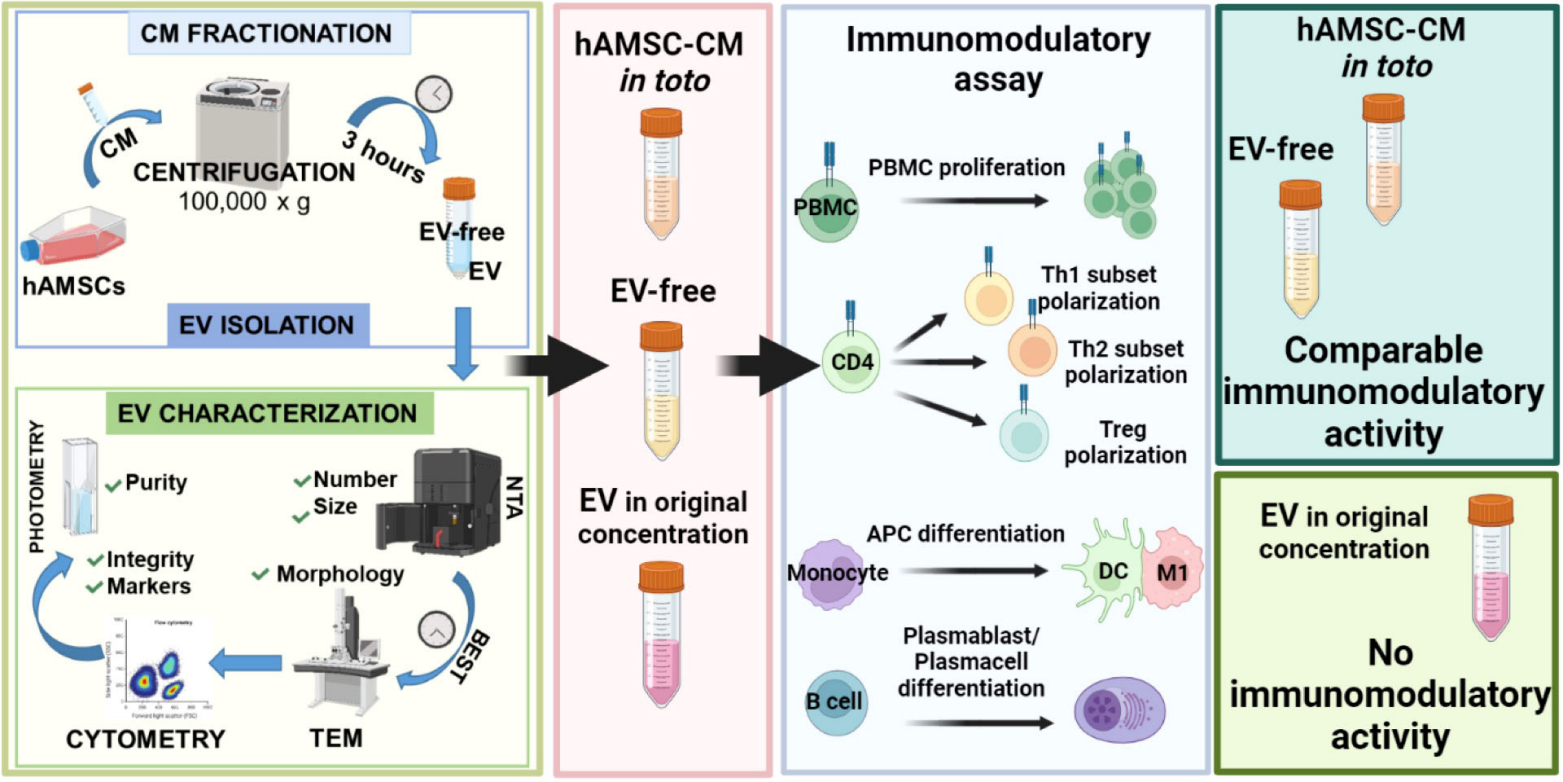
Summary figure hAMSC secrete factors able to modulate the immune system. This study demonstrates that the EV fraction of the hAMSC secretome has a negligible immunomodulatory effect while the EV-free fraction is the active component.

Our study demonstrates that EVs are not responsible for the immunomodulatory activity of the hAMSC secretome. For the first time, we compare EV-enriched and EV-depleted fractions obtained from the hAMSC secretome and demonstrate that the immunomodulatory effect is exerted by secreted factors not conveyed by EVs (**Figure 7**). Many factors have been reported to contribute to the immunomodulatory properties of MSC, such as the TNF-α-induced gene/protein 6 (TSG-6) (53), IL-10 (54), indoleamine 2,3-dioxygenase (IDO) (55), and inducible nitric-oxide (NO)-synthase (iNOS) (56). Also, hepatocyte growth factor (HGF) (57, 58), transforming growth factor β (TGF-β) (59, 60), and prostaglandin E2 (PGE2) (25, 61) are some of the most studied. However, the secretome represents a repertoire of cytokines, chemokines, and growth factors that combined together can act synergistically to contribute to the immunomodulatory activity of MSC not only *in vitro* but also *in vivo*. Indeed, many studies report the ability of MSC to contribute to regenerative processes in inflammatory-mediated diseases by modulating the immune response (62). Furthermore, MSCs are now being used in numerous clinical trials to treat diseases where a dysregulated immune system plays a major role in pathogenesis (63).

Finally, our findings suggest the utilization of the hAMSC secretome in toto when a modulation of immune response is desired. In addition, these data open a new possible scenario for the clinical application of the hAMSC secretome in toto that indeed contains two fractions that target either inflammatory immune cells or also other cell types involved in tissue regeneration, a combination of potentially synergistic effects that are highly desirable for an effective regenerative medicine strategy. A further not secondary advantage of hAMSC secretome in toto is the use of a less manipulated product which could contain costs and variability

## Data availability statement

The raw data supporting the conclusions of this article will be made available by the authors, without undue reservation. The datasets generated for this study can be found in the OSF Repository (https://osf.io/3ejxs/?view_only=40b9a42079ff43059d9236c56f61d465).

## Author contributions

Designing research study: AP, ER, LDG, and OP. Conducting experiments and acquiring data: EV, PR, AM, CPO, PBS. Analyzing data: AP, ER, AC, MM. Result interpretation: AP, ER, AC, MM, ARS. Writing and reviewing the manuscript: AP, ER, AC, ARS, LDG, OP. Financial support: LDG, OP. Final approval of the manuscript: LDG, OP. All authors contributed to the article and approved the submitted version.

## Funding

This work was supported by Ministero della Salute (Ricerca Corrente), Italian Ministry of Research and University (MIUR, 5x1000), PRIN 2017 program of the Italian Ministry of Research and University (MIUR, grant no. 2017RSAFK7), and Contributi per il funzionamento degli Enti privati che svolgono attività di ricerca - C.E.P.R. (2020-2021). Italian Ministry of Health, RCR-2021-23671217 project, under the The Italian Musculoskeletal Apparatus Network RAMS.

## Acknowledgments

The authors thank the physicians and midwives of the Department of Obstetrics and Gynecology of Fondazione Poliambulanza, Brescia, Italy, and all of the mothers who donated placenta. This work contributes to the COST Action CA17116 International Network for Translating Research on Perinatal Derivatives into Therapeutic Approaches (SPRINT), supported by COST (European Cooperation in Science and Technology).

## Conflict of interest

The authors declare that the research was conducted in the absence of any commercial or financial relationships that could be construed as a potential conflict of interest.

## Publisher’s note

All claims expressed in this article are solely those of the authors and do not necessarily represent those of their affiliated organizations, or those of the publisher, the editors and the reviewers. Any product that may be evaluated in this article, or claim that may be made by its manufacturer, is not guaranteed or endorsed by the publisher.

## Abbreviations

ANOVA, analysis of variance; AT, adipose tissue; BM, bone marrow; CFSE, carboxyfluorescein succinimidyl ester; CM, conditioned medium; DC, dendritic cell; Edu, 5-ethynyl-2′-deoxyuridine; EV, extracellular vesicles; GM-CSF, granulocyte-macrophage colony-stimulating factor; hAMSC, mesenchymal stromal cells derived from amniotic membrane; IFN-γ, interferon gamma; IL-4, interleukin-4; LPS, lipopolysaccharide; mAB, monoclonal antibody; mDC, mature dendritic cell; MFI, mean fluorescence intensity; Mo, monocyte; ns, not significant; NTA, nanoparticle tracking analysis; PBMC, peripheral blood mononuclear cells; PBS, phosphate buffered saline; P/S, penicillin/streptomycin; RPMI, Roswell Park Memorial Institute 1640 medium; RT, room temperature; Th, T helper cell; Treg, T regulatory cell; TEM, transmission electron microscopy; UC, umbilical cord.

## References

[1] Silini AR Masserdotti A Papait A Parolini O. Shaping the future of perinatal cells: Lessons from the past and interpretations of the present. Front Bioeng Biotechnol (2019) 7:75. doi: 10.3389/fbioe.2019.00075 31024907 PMC6467938

[2] Magatti M Vertua E Cargnoni A Silini A Parolini O. The immunomodulatory properties of amniotic cells: The two sides of the coin. Cell transplantation. (2018) 27(1):31–44. doi: 10.1177/0963689717742819 29562786 PMC6434482

[3] Silini AR Magatti M Cargnoni A Parolini O. Is immune modulation the mechanism underlying the beneficial effects of amniotic cells and their derivatives in regenerative medicine? Cell Transplant (2017) 26(4):531–9. doi: 10.3727/096368916X693699 PMC5661217 27938500

[4] Pianta S Bonassi Signoroni P Muradore I Rodrigues MF Rossi D Silini A. Amniotic membrane mesenchymal cells-derived factors skew T cell polarization toward treg and downregulate Th1 and Th17 cells subsets. Stem Cell Rev (2015) 11(3):394–407. doi: 10.1007/s12015-014-9558-4 PMC4451472 25348066

[5] Papait A Vertua E Magatti M. Mesenchymal stromal cells from fetal and maternal placenta possess key similarities and differences: Potential implications for their applications in regenerative medicine. (2020) 9(1):1–23. doi: 10.3390/cells9010127 PMC7017205 31935836

[6] Silini AR Papait A Cargnoni A Vertua E Romele P Bonassi Signoroni P. CM from intact hAM: an easily obtained product with relevant implications for translation in regenerative medicine. Stem Cell Res Ther (2021) 12(1):540. doi: 10.1186/s13287-021-02607-z 34641958 PMC8513276

[7] Magatti M Vertua E De Munari S Caro M Caruso M Silini A. Human amnion favours tissue repair by inducing the M1-to-M2 switch and enhancing M2 macrophage features. J Tissue Eng regenerative Med (2017) 11(10):2895–911. doi: 10.1002/term.2193 PMC5697700 27396853

[8] Magatti M De Munari S Vertua E Nassauto C Albertini A Wengler GS. Amniotic mesenchymal tissue cells inhibit dendritic cell differentiation of peripheral blood and amnion resident monocytes. Cell transplantation. (2009) 18(8):899–914. doi: 10.3727/096368909X471314 19523334

[9] Magatti M Masserdotti A Bonassi Signoroni P Vertua E Stefani FR Silini AR. B lymphocytes as targets of the immunomodulatory properties of human amniotic mesenchymal stromal cells. Front Immunol (2020) 11:1156. doi: 10.3389/fimmu.2020.01156 32582218 PMC7295987

[10] Cargnoni A Piccinelli EC Ressel L Rossi D Magatti M Toschi I. Conditioned medium from amniotic membrane-derived cells prevents lung fibrosis and preserves blood gas exchanges in bleomycin-injured mice-specificity of the effects and insights into possible mechanisms. Cytotherapy (2014) 16(1):17–32. doi: 10.1016/j.jcyt.2013.07.002 24094500

[11] Cargnoni A Romele P Bonassi Signoroni P Farigu S Magatti M Vertua E. Amniotic MSCs reduce pulmonary fibrosis by hampering lung b-cell recruitment, retention, and maturation. Stem Cells Trans Med (2020) 9(9):1023–35. doi: 10.1002/sctm.20-0068 PMC7445028 32452646

[12] Lee PH Tu CT Hsiao CC Tsai MS Ho CM Cheng NC. Antifibrotic activity of human placental amnion membrane-derived CD34+ mesenchymal Stem/Progenitor cell transplantation in mice with thioacetamide-induced liver injury. Stem Cells Trans Med (2016) 5(11):1473–84. doi: 10.5966/sctm.2015-0343 PMC5070504 27405780

[13] Kim SW Zhang HZ Guo L Kim JM Kim MH. Amniotic mesenchymal stem cells enhance wound healing in diabetic NOD/SCID mice through high angiogenic and engraftment capabilities. PLoS One (2012) 7(7):e41105. doi: 10.1371/journal.pone.0041105 22815931 PMC3398889

[14] Parolini O Souza-Moreira L O’Valle F Magatti M Hernandez-Cortes P Gonzalez-Rey E. Therapeutic effect of human amniotic membrane-derived cells on experimental arthritis and other inflammatory disorders. Arthritis Rheumatol (2014) 66(2):327–39. doi: 10.1002/art.38206 24504805

[15] Zhang J Liu Z Tang J Li Y You Q Yang J. Fibroblast growth factor 2-induced human amniotic mesenchymal stem cells combined with autologous platelet rich plasma augmented tendon-to-bone healing. J orthopaedic translation. (2020) 24:155–65. doi: 10.1016/j.jot.2020.01.003 PMC7548348 33101966

[16] Jiang F Zhang W Zhou M Zhou Z Shen M Chen N. Human amniotic mesenchymal stromal cells promote bone regeneration *via* activating endogenous regeneration. Theranostics (2020) 10(14):6216–30. doi: 10.7150/thno.45249 PMC7255030 32483449

[17] Onishi R Ohnishi S Higashi R Watari M Yamahara K Okubo N. Human amnion-derived mesenchymal stem cell transplantation ameliorates dextran sulfate sodium-induced severe colitis in rats. Cell Transplantation (2015) 24(12):2601–14. doi: 10.3727/096368915X687570 25812083

[18] Pischiutta F Brunelli L Romele P Silini A Sammali E Paracchini L. Protection of brain injury by amniotic mesenchymal stromal cell-secreted metabolites. Crit Care Med (2016) 44(11):e1118–e31. doi: 10.1097/CCM.0000000000001864 27441900

[19] Giampà C Alvino A Magatti M Silini AR Cardinale A Paldino E. Conditioned medium from amniotic cells protects striatal degeneration and ameliorates motor deficits in the R6/2 mouse model of huntington’s disease. J Cell Mol Med (2019) 23(2):1581–92. doi: 10.1111/jcmm.14113 PMC6349233 30585395

[20] Yáñez-Mó M Siljander PR Andreu Z Zavec AB Borràs FE Buzas EI. Biological properties of extracellular vesicles and their physiological functions. J Extracellular Vesicles (2015) 4:27066. doi: 10.3402/jev.v4.27066 25979354 PMC4433489

[21] Seo Y Kim HS. Stem cell-derived extracellular vesicles as immunomodulatory therapeutics. Stem Cells Int (2019) 2019:5126156. doi: 10.1155/2019/5126156 30936922 PMC6413386

[22] Keshtkar S Azarpira N Ghahremani MH. Mesenchymal stem cell-derived extracellular vesicles: novel frontiers in regenerative medicine. Stem Cell Res Ther (2018) 9(1):63. doi: 10.1186/s13287-018-0791-7 29523213 PMC5845209

[23] Ragni E Papait A Perucca Orfei C. Amniotic membrane-mesenchymal stromal cells secreted factors and extracellular vesicle-miRNAs: Anti-inflammatory and regenerative features for musculoskeletal tissues. Stem Cells Transl Med (2021) 10(7):1044–62. doi: 10.1002/sctm.20-0390 PMC8235131 33656805

[24] Magatti M Pianta S Silini A Parolini O. Isolation, culture, and phenotypic characterization of mesenchymal stromal cells from the amniotic membrane of the human term placenta. Methods Mol Biol (Clifton NJ) (2016) 1416:233–44. doi: 10.1007/978-1-4939-3584-0_13 27236675

[25] Rossi D Pianta S Magatti M Sedlmayr P Parolini O. Characterization of the conditioned medium from amniotic membrane cells: prostaglandins as key effectors of its immunomodulatory activity. PLoS One (2012) 7(10):e46956. doi: 10.1371/journal.pone.0046956 23071674 PMC3468614

[26] Pianta S Magatti M Vertua E Bonassi Signoroni P Muradore I Nuzzo AM. Amniotic mesenchymal cells from pre-eclamptic placentae maintain immunomodulatory features as healthy controls. J Cell Mol Med (2016) 20(1):157–69. doi: 10.1111/jcmm.12715 PMC4717851 26515425

[27] Wingender G Kronenberg M. OMIP-030: Characterization of human T cell subsets *via* surface markers. Cytometry Part A: J Int Soc Analytical Cytology (2015) 87(12):1067–9. doi: 10.1002/cyto.a.22788 26506224

[28] Théry C Witwer KW. Minimal information for studies of extracellular vesicles 2018 (MISEV2018): a position statement of the international society for extracellular vesicles and update of the MISEV2014 guidelines. J Extracellular Vesicles (2018) 7(1):1535750. 30637094 10.1080/20013078.2018.1535750 PMC6322352

[29] Webber J Clayton A. How pure are your vesicles? J Extracellular Vesicles (2013) 2:1–6. doi: 10.3402/jev.v2i0.19861 PMC3760653 24009896

[30] Cargnoni A Papait A Masserdotti A Pasotti A Stefani FR Silini AR. Extracellular vesicles from perinatal cells for anti-inflammatory therapy. Front Bioengineering Biotechnol (2021) 9:637737. doi: 10.3389/fbioe.2021.637737 PMC7892960 33614619

[31] Di Trapani M Bassi G Midolo M Gatti A Kamga PT Cassaro A. Differential and transferable modulatory effects of mesenchymal stromal cell-derived extracellular vesicles on T, b and NK cell functions. Sci Rep (2016) 6:24120. doi: 10.1038/srep24120 27071676 PMC4829861

[32] Conforti A Scarsella M Starc N Giorda E Biagini S Proia A. Microvescicles derived from mesenchymal stromal cells are not as effective as their cellular counterpart in the ability to modulate immune responses in vitro. Stem Cells Dev (2014) 23(21):2591–9. doi: 10.1089/scd.2014.0091 PMC4201301 24937591

[33] Lange-Consiglio A Perrini C Tasquier R Deregibus MC Camussi G Pascucci L. Equine amniotic microvesicles and their anti-inflammatory potential in a tenocyte model in vitro. Stem Cells Dev (2016) 25(8):610–21. doi: 10.1089/scd.2015.0348 26914245

[34] Franco da Cunha F Andrade-Oliveira V Candido de Almeida D Borges da Silva T Naffah de Souza Breda C Costa Cruz M. Extracellular vesicles isolated from mesenchymal stromal cells modulate CD4(+) T lymphocytes toward a regulatory profile. Cells (2020) 9(4):1–27. doi: 10.3390/cells9041059 PMC7226573 32340348

[35] Gouveia de Andrade AV Bertolino G Riewaldt J Bieback K Karbanová J Odendahl M. Extracellular vesicles secreted by bone marrow- and adipose tissue-derived mesenchymal stromal cells fail to suppress lymphocyte proliferation. Stem Cells Dev (2015) 24(11):1374–6. doi: 10.1089/scd.2014.0563 25779336

[36] Del Fattore A Luciano R Pascucci L Goffredo BM Giorda E Scapaticci M. Immunoregulatory effects of mesenchymal stem cell-derived extracellular vesicles on T lymphocytes. Cell Transplantation (2015) 24(12):2615–27. doi: 10.3727/096368915X687543 25695896

[37] Budoni M Fierabracci A Luciano R Petrini S Di Ciommo V Muraca M. The immunosuppressive effect of mesenchymal stromal cells on b lymphocytes is mediated by membrane vesicles. Cell Transplantation (2013) 22(2):369–79. doi: 10.3727/096368911X582769b 23433427

[38] Blazquez R Sanchez-Margallo FM de la Rosa O Dalemans W Alvarez V Tarazona R. Immunomodulatory potential of human adipose mesenchymal stem cells derived exosomes on *in vitro* stimulated T cells. Front Immunol (2014) 5:556. doi: 10.3389/fimmu.2014.00556 25414703 PMC4220146

[39] Lopatina T Favaro E Grange C Cedrino M Ranghino A Occhipinti S. PDGF enhances the protective effect of adipose stem cell-derived extracellular vesicles in a model of acute hindlimb ischemia. Sci Rep (2018) 8(1):17458. doi: 10.1038/s41598-018-36143-3 30514962 PMC6279818

[40] Carreras-Planella L Monguió-Tortajada M Borràs FE Franquesa M. Immunomodulatory effect of MSC on b cells is independent of secreted extracellular vesicles. Front Immunol (2019) 10:1288. doi: 10.3389/fimmu.2019.01288 31244839 PMC6563675

[41] Kim H Lee MJ Bae EH Ryu JS Kaur G Kim HJ. Comprehensive molecular profiles of functionally effective MSC-derived extracellular vesicles in immunomodulation. Mol therapy: J Am Soc Gene Ther (2020) 28(7):1628–44. doi: 10.1016/j.ymthe.2020.04.020 PMC7335740 32380062

[42] Burrello J Monticone S Gai C Gomez Y Kholia S Camussi G. Stem cell-derived extracellular vesicles and immune-modulation. Front Cell Dev Biol (2016) 4:83. doi: 10.3389/fcell.2016.00083 27597941 PMC4992732

[43] Witwer KW Buzás EI Bemis LT Bora A Lässer C Lötvall J. Standardization of sample collection, isolation and analysis methods in extracellular vesicle research. J Extracellular Vesicles (2013) 2:1–25. doi: 10.3402/jev.v2i0.20360 PMC3760646 24009894

[44] Gardiner C Di Vizio D Sahoo S Théry C Witwer KW Wauben M. Techniques used for the isolation and characterization of extracellular vesicles: results of a worldwide survey. J Extracellular Vesicles (2016) 5:32945. doi: 10.3402/jev.v5.32945 27802845 PMC5090131

[45] Whittaker TE Nagelkerke A Nele V Kauscher U Stevens MM. Experimental artefacts can lead to misattribution of bioactivity from soluble mesenchymal stem cell paracrine factors to extracellular vesicles. J Extracell Vesicles (2020) 9(1):1807674. doi: 10.1080/20013078.2020.1807674 32944192 PMC7480412

[46] Bobrie A Théry C. Exosomes and communication between tumours and the immune system: are all exosomes equal? Biochem Soc Trans (2013) 41(1):263–7. doi: 10.1042/BST20120245 23356294

[47] Kowal J Arras G Colombo M Jouve M Morath JP Primdal-Bengtson B. Proteomic comparison defines novel markers to characterize heterogeneous populations of extracellular vesicle subtypes. Proc Natl Acad Sci USA (2016) 113(8):E968–77. doi: 10.1073/pnas.1521230113 PMC4776515 26858453

[48] Zhang H Freitas D Kim HS Fabijanic K Li Z Chen H. Identification of distinct nanoparticles and subsets of extracellular vesicles by asymmetric flow field-flow fractionation. Nat Cell Biol (2018) 20(3):332–43. doi: 10.1038/s41556-018-0040-4 PMC5931706 29459780

[49] Lange-Consiglio A Funghi F Cantile C Idda A Cremonesi F Riccaboni P. Case report: Use of amniotic microvesicles for regenerative medicine treatment of a mare with chronic endometritis. Front Veterinary Science (2020) 7:347. doi: 10.3389/fvets.2020.00347 PMC7311574 32626730

[50] Ohara M Ohnishi S. Extracellular vesicles from amnion-derived mesenchymal stem cells ameliorate hepatic inflammation and fibrosis in rats. Stem Cells Int (2018) 2018:321264. doi: 10.1155/2018/3212643 PMC6323530 30675167

[51] Zavatti M Beretti F Casciaro F Bertucci E Maraldi T. Comparison of the therapeutic effect of amniotic fluid stem cells and their exosomes on monoiodoacetate-induced animal model of osteoarthritis. BioFactors (Oxford England) (2020) 46(1):106–17. doi: 10.1002/biof.1576 31625201

[52] Perrini C Strillacci MG Bagnato A Esposti P Marini MG Corradetti B. Microvesicles secreted from equine amniotic-derived cells and their potential role in reducing inflammation in endometrial cells in an in-vitro model. Stem Cell Res Ther (2016) 7(1):169. doi: 10.1186/s13287-016-0429-6 27863532 PMC5114748

[53] Lo Sicco C Reverberi D Balbi C Ulivi V Principi E Pascucci L. Mesenchymal stem cell-derived extracellular vesicles as mediators of anti-inflammatory effects: Endorsement of macrophage polarization. Stem Cells Trans Med (2017) 6(3):1018–28. doi: 10.1002/sctm.16-0363 PMC5442783 28186708

[54] Abumaree MH Al Jumah MA Kalionis B Jawdat D Al Khaldi A Abomaray FM. Human placental mesenchymal stem cells (pMSCs) play a role as immune suppressive cells by shifting macrophage differentiation from inflammatory M1 to anti-inflammatory M2 macrophages. Stem Cell Rev Rep (2013) 9(5):620–41. doi: 10.1007/s12015-013-9455-2 23812784

[55] Opitz CA Litzenburger UM Lutz C Lanz TV Tritschler I Köppel A. Toll-like receptor engagement enhances the immunosuppressive properties of human bone marrow-derived mesenchymal stem cells by inducing indoleamine-2,3-dioxygenase-1 *via* interferon-beta and protein kinase r. Stem Cells (Dayton Ohio) (2009) 27(4):909–19. doi: 10.1002/stem.7 19353519

[56] Maria ATJ Rozier P Fonteneau G Sutra T Maumus M Toupet K. iNOS activity is required for the therapeutic effect of mesenchymal stem cells in experimental systemic sclerosis. Front Immunol (2018) 9:3056. doi: 10.3389/fimmu.2018.03056 30622540 PMC6308989

[57] Deng Y Zhang Y Ye L Zhang T Cheng J Chen G. Umbilical cord-derived mesenchymal stem cells instruct monocytes towards an IL10-producing phenotype by secreting IL6 and HGF. Sci Rep (2016) 6:37566. doi: 10.1038/srep37566 27917866 PMC5137158

[58] Melief SM Geutskens SB Fibbe WE Roelofs H. Multipotent stromal cells skew monocytes towards an anti-inflammatory interleukin-10-producing phenotype by production of interleukin-6. Haematologica (2013) 98(6):888–95. doi: 10.3324/haematol.2012.078055 PMC3669444 23349310

[59] Melief SM Schrama E Brugman MH Tiemessen MM Hoogduijn MJ Fibbe WE. Multipotent stromal cells induce human regulatory T cells through a novel pathway involving skewing of monocytes toward anti-inflammatory macrophages. Stem Cells (Dayton Ohio) (2013) 31(9):1980–91. doi: 10.1002/stem.1432 23712682

[60] Ko JH Lee HJ Jeong HJ Kim MK Wee WR Yoon SO. Mesenchymal stem/stromal cells precondition lung monocytes/macrophages to produce tolerance against allo- and autoimmunity in the eye. Proc Natl Acad Sci USA (2016) 113(1):158–63. doi: 10.1073/pnas.1522905113 PMC4711840 26699483

[61] Wang J Liu Y Ding H Shi X Ren H. Mesenchymal stem cell-secreted prostaglandin E(2) ameliorates acute liver failure *via* attenuation of cell death and regulation of macrophage polarization. Stem Cell Res Ther (2021) 12(1):15. doi: 10.1186/s13287-020-02070-2 33413632 PMC7792134

[62] Klinker MW Wei CH. Mesenchymal stem cells in the treatment of inflammatory and autoimmune diseases in experimental animal models. World J Stem Cells (2015) 7(3):556–67. doi: 10.4252/wjsc.v7.i3.556 PMC4404391 25914763

[63] Wang LT Ting CH Yen ML Liu KJ Sytwu HK Wu KK. Human mesenchymal stem cells (MSCs) for treatment towards immune- and inflammation-mediated diseases: review of current clinical trials. J Biomed Science (2016) 23(1):76. doi: 10.1186/s12929-016-0289-5 PMC5095977 27809910

