# Supplementary material for: Comparison of EV-free fraction, EVs, and total secretome of amniotic mesenchymal stromal cells for their immunomodulatory potential: a translational perspective
Source: Front Immunol. 2022 Aug 16;13:960909. doi: 10.3389/fimmu.2022.960909 (PMC9424831; doi:10.3389/fimmu.2022.960909)
Supplement: Supplementary file 1 [file DataSheet_1.docx]

Supplementary Material

# Supplementary Figures and Tables

**
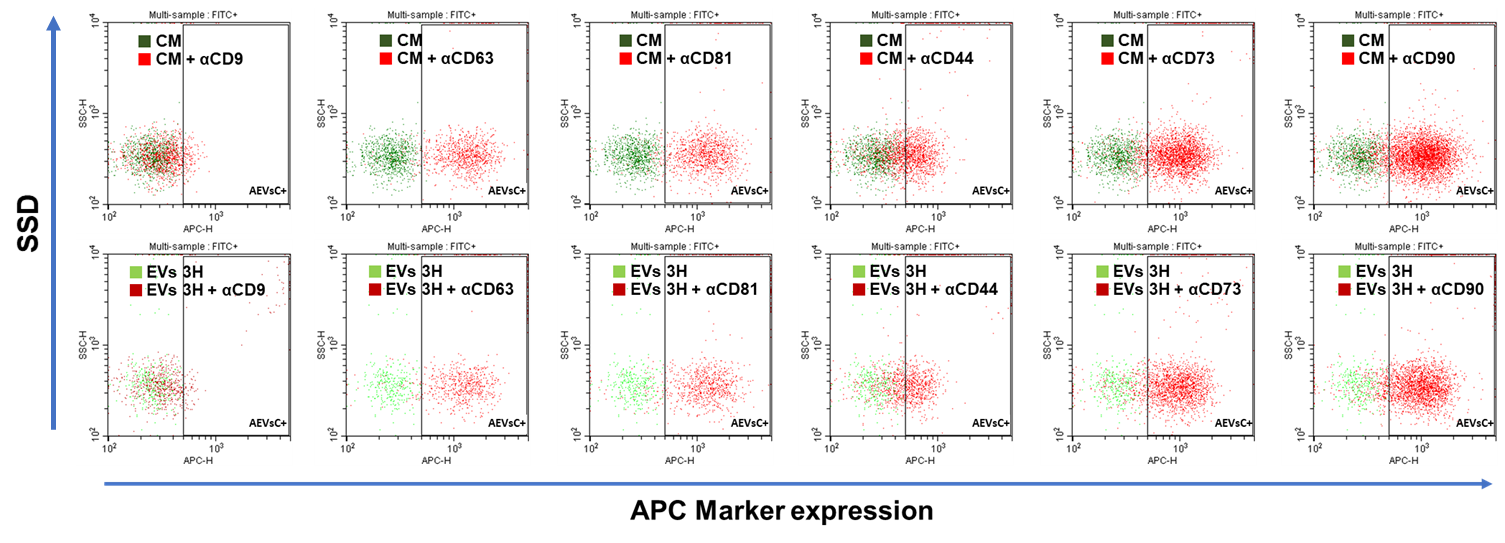
** **Supplementary figure 1)** EV-markers flow cytometry analysis in the unprocessed CM or in the pellets (P) after 3-hours centrifugation. After EVs gating in the FITC channel as per panel F, EVs showed positive staining for CD63/81 EV-related molecules and CD44/73/90 MSC markers. CD9, another EV postulated marker, was barely detectable. Representative cytograms are presented*.*

.
